# Comparative analysis of unilateral biportal endoscopy and minimally invasive transforaminal lumbar interbody fusion in treating lumbar spinal brucellosis

**DOI:** 10.3389/fsurg.2026.1830438

**Published:** 2026-06-01

**Authors:** Cao Rui, Yin Ming

**Affiliations:** 1 Department of Spinal Surgery, The First Affiliated Hospital of Xinjiang Medical University, Urumqi, Xinjiang Uygur Autonomous Region, China; 2 Department of Orthopedics and Traumatology, Wuxi Hospital of Traditional Chinese Medicine, Wuxi, Jiangsu, China

**Keywords:** clinical efficacy, lumbar spinal brucellosis, minimally invasive surgery, minimally invasive transforaminal lumbar interbody fusion, unilateral biportal endoscopy

## Abstract

**Objective:**

This study aimed to compare the clinical efficacy and safety of unilateral biportal endoscopy-assisted transforaminal lumbar interbody fusion (UBE-TLIF) vs. minimally invasive transforaminal lumbar interbody fusion (MIS-TLIF) in the treatment of lumbar spinal brucellosis, with the goal of identifying an optimized surgical strategy.

**Methods:**

A retrospective analysis was conducted on 62 patients with lumbar spinal brucellosis who underwent surgery between January 2018 and June 2024. Patients were divided into two groups based on the surgical approach: UBE-TLIF (*n* = 32) and MIS-TLIF (*n* = 30). Baseline characteristics, perioperative parameters (operative time, estimated blood loss, incision length, hospital stay), inflammatory markers [Brucella agglutination titer, erythrocyte sedimentation rate [ESR], C-reactive protein [CRP], procalcitonin [PCT]], pain scores [visual analog scale (VAS) for back and leg pain], functional outcomes [Oswestry Disability Index (ODI)], intervertebral disc height, complications, bone fusion rate, and MacNab criteria were compared between groups.

**Results:**

The two groups were comparable in baseline characteristics (*P* > 0.05). The UBE-TLIF group demonstrated significantly shorter operative time and hospital stay, less intraoperative blood loss, and smaller incisions (*P* < 0.05). Postoperative inflammatory markers decreased significantly in both groups (*P* < 0.05), with no significant intergroup differences (*P* > 0.05). The UBE-TLIF group had significantly lower VAS scores at 1 and 3 months postoperatively (*P* < 0.05), but these differences resolved by 6 months and final follow-up (*P* > 0.05). Both groups showed significant improvements in ODI and intervertebral disc height postoperatively (*P* < 0.05), with no intergroup differences (*P* > 0.05). Complications included three dural tears (UBE-TLIF) and four nerve root injuries (MIS-TLIF), all managed conservatively, with comparable complication rates (*P* > 0.05). Mean follow-up was 18.5 ± 3.6 months in the UBE-TLIF group and 18.1 ± 3.9 months in the MIS-TLIF group (*P* > 0.05). No recurrence or implant-related complications occurred. Bone fusion rates (96.88% vs. 93.33%) and MacNab excellent/good rates (93.75% vs. 90.00%) were similar between groups (*P* > 0.05).

**Conclusion:**

Both UBE-TLIF and MIS-TLIF are safe and effective surgical options for lumbar spinal brucellosis, providing adequate infection control, pain relief, functional restoration, and satisfactory bone fusion. UBE-TLIF offers superior minimally invasive benefits, including shorter operative time, reduced blood loss, less surgical trauma, and enhanced early postoperative pain control, supporting its preferential application in appropriately selected patients by surgeons with relevant expertise.

## 1 Introduction

Brucellosis is a zoonotic infectious disease caused by Brucella infection, and spinal brucellosis is one of the most common musculoskeletal system complications, with the lumbar spine as the most frequently affected segment (1, 2). Lumbar spinal brucellosis is mainly characterized by vertebral bone destruction, intervertebral space narrowing, inflammatory reaction and even spinal canal stenosis and nerve root compression, which can cause persistent low back and leg pain, motor and sensory dysfunction of lower limbs, and seriously affect the quality of life of patients if not treated in time (3). At present, the clinical treatment of lumbar spinal brucellosis is based on standardized anti-brucellosis drug therapy, and surgical intervention is required for patients with obvious vertebral bone destruction, spinal instability, nerve compression symptoms and ineffective conservative treatment (4).

The traditional open surgical methods for lumbar spinal brucellosis have the disadvantages of large trauma, large intraoperative blood loss, long postoperative recovery time and high incidence of complications, which limit their clinical application to a certain extent (5). With the rapid development of minimally invasive spinal surgery technology, Minimally Invasive Transforaminal Lumbar Interbody Fusion (MIS-TLIF) and Unilateral Biportal Endoscopy-Assisted Transforaminal Lumbar Interbody Fusion (UBE-TLIF) have been gradually applied to the treatment of spinal infectious diseases due to their advantages of minimal trauma, precise operation and rapid postoperative recovery (6, 7). MIS-TLIF realizes transforaminal lumbar interbody fusion through the Wiltse paraspinal muscle approach, which can reduce the damage to paraspinal muscles and maintain the stability of the posterior spinal structure, but its surgical field of view is relatively limited, which may lead to incomplete debridement of focus in the case of extensive lumbar spinal brucellosis lesions, and the operation has high requirements for the surgical skills of doctors (8).

UBE technique is an emerging minimally invasive spinal surgery method, which has been used in recent years to treat various degenerative spinal diseases. However, there are no relevant reports in the literature on the use of this technique to treat lumbar spinal brucellosis (9, 10). However, the clinical application of UBE-TLIF in lumbar spinal brucellosis is still in the exploratory stage, and there are few comparative studies with MIS-TLIF on its efficacy and safety.

In view of this, this study expanded the sample size and conducted a retrospective comparative analysis of the clinical data of patients with lumbar spinal brucellosis treated with UBE-TLIF and MIS-TLIF, focusing on comparing the differences in surgical indicators, inflammatory control, pain relief, functional recovery, complications and long-term efficacy between the two surgical methods, in order to provide more sufficient clinical evidence for the selection of optimized surgical schemes for lumbar spinal brucellosis.

## 2 Materials and methods

### 2.1 General information

A retrospective analysis was conducted on the clinical data of patients with lumbar spinal brucellosis who underwent surgical treatment in our hospital from January 2018 to June 2024. The study was approved by the Medical Ethics Committee of our hospital, and all patients or their family members signed the informed consent form.The allocation of surgical techniques was determined based on a combination of surgeon expertise and patient-specific factors. UBE-TLIF was primarily selected for patients with relatively localized lesions requiring meticulous manipulation in the spinal canal and intervertebral foramen region, and was performed by surgeons proficient in endoscopic techniques. MIS-TLIF was preferentially selected for patients with more extensive lesions requiring a wider surgical field, or when the surgeon had relatively less endoscopic experience. All surgical decisions were made preoperatively by the same surgical team based on imaging characteristics, lesion extent, and the surgeon's technical proficiency, aiming to minimize selection bias to the greatest extent possible within the constraints of a retrospective design.

#### 2.1.1 Inclusion Criteria

(1) The diagnosis of lumbar spinal brucellosis was confirmed by clinical symptoms, laboratory examinations (positive brucella agglutination test), imaging examinations (x-ray, CT, MRI showing vertebral bone destruction, intervertebral space narrowing, inflammatory infiltration) and pathological examination (if necessary); (2) The affected segment was single-segment lumbar spine (L_1_-L_5_); (3) Standardized anti-brucellosis drug treatment was given for more than 4 weeks before surgery, and the inflammatory indicators were in the declining stage but the clinical symptoms were not relieved; (4) There were indications for surgery: obvious vertebral bone destruction, spinal instability, spinal canal stenosis/nerve root compression with lower limb motor and sensory dysfunction, or persistent severe low back and leg pain; (5) No serious organic diseases of heart, liver, kidney and other important organs, and able to tolerate surgery; (6) Complete clinical and follow-up data.

#### 2.1.2 Exclusion Criteria

(1) Patients with active systemic brucellosis or combined with other infectious diseases; (2) Multi-segment lumbar spinal brucellosis or combined with thoracic/cervical spinal brucellosis; (3) Severe osteoporosis, spinal tumor, spinal trauma and other diseases leading to vertebral bone destruction; (4) Severe spinal canal stenosis with spinal cord injury and paraplegia; (5) Patients with coagulation dysfunction, immune deficiency and other surgical contraindications; (6) Pregnant and lactating women; (7) Loss to follow-up or incomplete clinical data.

According to the above criteria, a total of 62 patients were included in the study, including 38 males and 24 females, aged 32–64 years old, with a disease course of 2–10 months. According to the different surgical methods, they were divided into the UBE-TLIF group (32 cases) and the MIS-TLIF group (30 cases).

#### 2.1.3 Sample size estimation

A *post-hoc* power analysis was performed using G Power software (version 3.1.9.7) based on the primary outcome indicator of operative time. Assuming a two-sided significance level (*α*) of 0.05, a power (1-*β*) of 0.80, and an effect size (Cohen's d) of 0.75 derived from the observed difference in operative time between the two groups (95.25 ± 15.68 min vs. 138.67 ± 20.35 min), the required sample size was calculated to be approximately 28 patients per group. The actual sample sizes of 32 in the UBE-TLIF group and 30 in the MIS-TLIF group therefore meet the requirement for detecting a clinically meaningful difference in the primary outcome. However, for secondary outcomes such as complication rates with lower expected incidence, the sample size may be insufficient to detect small differences, and caution should be exercised in interpreting these findings.

### 2.2 Preoperative management

All patients received standardized oral anti-brucellosis drug treatment for more than 4 weeks before surgery, including doxycycline, rifampicin and streptomycin (adjusted according to the patient's renal function and drug sensitivity test). During the preoperative period, the patients’ inflammatory indicators (brucella agglutination titer, ESR, CRP, PCT) were regularly rechecked, and the nutritional status of the patients was improved by nutritional support treatment for those with malnutrition. Surgery was performed only when the clinical symptoms of the patients were stable, the inflammatory indicators were significantly decreased and no longer rising, and the general condition was good and able to tolerate surgery. Preoperative routine imaging examination (lumbar spine x-ray, thin-layer CT, MRI) was completed to clarify the location of the affected segment, the scope of bone destruction, the degree of spinal canal stenosis and nerve root compression.

### 2.3 Surgical methods

All surgeries were performed by a team of three senior spinal surgeons, each with over 8 years of experience in minimally invasive spinal surgery. The two primary techniques were distributed among the surgeons as follows: UBE-TLIF was performed by two surgeons, each having completed over 50 independent UBE-TLIF cases prior to this study; MIS-TLIF was performed by all three surgeons, with each having completed over 80 independent MIS-TLIF cases prior to this study. For the purposes of this study, “proficiency” in UBE-TLIF was defined as completion of at least 40 independent cases, based on previously published learning curve analyses demonstrating that surgical time and complication rates stabilize after approximately 30–40 cases.All surgeons in this study exceeded this threshold, ensuring comparable technical proficiency across the two techniques and minimizing potential bias related to surgical experience.

#### 2.3.1 MIS-TLIF group

The patient was placed in the prone position, with abdominal suspension to reduce the pressure of the lumbar spine. C-arm x-ray machine was used for intraoperative positioning to confirm the affected lumbar segment. The Wiltse paraspinal muscle approach was adopted, and two longitudinal incisions of about 1.5–2.0 cm were made at 1.5 cm beside the midline of the back of the responsible segment. The paraspinal muscle was separated along the muscle space, and the pedicle of the responsible segment was exposed. Pedicle screws were implanted into the bilateral pedicles of the upper and lower vertebral bodies of the affected segment under C-arm fluoroscopy. The soft tissue attached to the lamina was stripped, the medial side of the lamina, lateral recess and intervertebral foramen were expanded with a lamina rongeur, and the brucellosis focus (necrotic bone tissue, inflammatory granulation tissue) in the spinal canal and intervertebral space was thoroughly debrided. The cartilage endplate of the intervertebral space was completely removed with a curette until the fresh bone surface was exposed to prepare the bone graft bed. The autologous bone graft (removed lamina bone and spinous process bone) was filled into the intervertebral space, and the appropriate size cage was implanted. The nail rod system was installed to fix and restore the height of the intervertebral space and the physiological curvature of the lumbar spine. The surgical field was repeatedly rinsed with normal saline containing rifampicin (2 g/1000 mL), the drainage tube was placed, and the incision was sutured layer by layer.

#### 2.3.2 UBE-TLIF group

The patient was placed in the prone position with abdominal suspension, and C-arm x-ray machine was used to locate the affected segment and mark the pedicle projection. Two longitudinal small incisions of about 0.8–1.0 cm were made along the pedicle projection of the upper and lower vertebral bodies adjacent to the affected segment, which were used as the endoscopic observation channel and the working channel respectively. The deep fascia was incised, the paraspinal muscle was separated along the muscle space, and the dilator was used to establish the channel, and the endoscopic sheath was placed. The arthroscope was connected to the observation channel, and the plasma radiofrequency knife, osteotome, curette and other surgical instruments were placed through the working channel. Under endoscopic vision, the superior and inferior articular processes and intervertebral foramen area of the affected segment were exposed, the inferior articular process and the tip of the superior articular process were partially resected with an osteotome, and the ligamentum flavum was removed to fully decompress the spinal canal and nerve root. The nerve root and dural sac were gently retracted medially with a nerve hook to expose the intervertebral space, and the brucellosis focus in the intervertebral space was thoroughly debrided under direct vision, including necrotic bone tissue, inflammatory granulation tissue and pus. The cartilage endplate was carefully treated to expose the fresh bone surface, and the autologous bone graft was filled into the intervertebral space and the cage was implanted. The surgical field was repeatedly irrigated with normal saline containing rifampicin (2 g/1,000 mL) to eliminate residual focus and inflammatory factors. Percutaneous pedicle screw implantation was performed under C-arm fluoroscopy, the nail rod system was installed for internal fixation, the drainage tube was placed, and the two small incisions were sutured respectively.

### 2.4 Postoperative management

After surgery, the patients were given anti-infection, analgesia, nutritional support and other symptomatic supportive treatment, and the drainage tube was removed when the drainage volume was less than 50 mL/d. The anti-brucellosis drug treatment was continued for 6–12 months according to the patient's condition, and the drug was adjusted according to the recheck results of inflammatory indicators and brucella agglutination titer. The patients were instructed to carry out functional exercise of lumbar spine and lower limbs step by step according to the postoperative recovery situation: bed exercise was started 24–48 h after surgery, sitting and standing exercise with waist support was started 1 week after surgery, and the waist support was removed for gradual lumbar spine activity 3 months after surgery, and heavy physical labor was prohibited within 1 year. Regular reexaminations were performed at 2 weeks, 1 month, 3 months, 6 months, 12 months after surgery and every 6 months thereafter, including laboratory examinations (brucella agglutination titer, ESR, CRP, PCT) and imaging examinations (lumbar spine x-ray, CT), to evaluate the inflammatory control, bone fusion and spinal stability.

### 2.5 Observation indicators and efficacy evaluation

#### 2.5.1 General and surgical indicators

Record the baseline data (gender, age, BMI, disease course, affected spinal segments) of the two groups; compare the surgical indicators including operative time, intraoperative blood loss, incision length and hospitalization time.

#### 2.5.2 Inflammatory indicators

Detect and compare the brucella agglutination titer, ESR, CRP and PCT of the two groups before surgery and at 2 weeks, 1 month, 3 months and 6 months after surgery.

#### 2.5.3 Pain and functional recovery indicators

Evaluate and compare the Visual analog scale(VAS) scores of low back and leg pain (0–10 points, the higher the score, the more severe the pain) and Oswestry Disability Index(ODI) (0%–100%, the higher the score, the worse the lumbar spine function) of the two groups before surgery and at 1 week, 1 month, 3 months, 6 months after surgery and the final follow-up.

#### 2.5.4 Spinal structural Index

Measure and compare the anterior and posterior intervertebral space height of the affected segment of the two groups before surgery and at the final follow-up by lumbar spine x-ray, and calculate the recovery rate of intervertebral space height.

#### 2.5.5 Postoperative complications

Record the occurrence of postoperative complications in the two groups, including dural sac tear, nerve root injury, cerebrospinal fluid leakage, incision infection, epidural hematoma, implant loosening and brucellosis recurrence, and calculate the total complication rate.

#### 2.5.6 Long-term efficacy indicators

(1) Bone fusion rate: evaluated by lumbar spine CT at the final follow-up, the bone fusion criteria were: the trabecular bone passed through the bone graft area, the intervertebral space was stable, no obvious cage subsidence or displacement, and no abnormal movement of the affected segment on dynamic x-ray; (2) MacNab efficacy evaluation: excellent (no low back and leg pain, normal lumbar spine activity, no impact on daily life and work), good (occasional mild low back and leg pain, slight limitation of lumbar spine activity, little impact on daily life and work), fair (frequent low back and leg pain, obvious limitation of lumbar spine activity, great impact on daily life and work), poor (persistent severe low back and leg pain, loss of lumbar spine activity function, unable to take care of oneself); the excellent and good rate = (excellent cases + good cases)/total cases × 100%.

### 2.6 Statistical methods

SPSS 26.0 statistical software was used for data analysis. The measurement data conforming to normal distribution were expressed as (*x̅* *±* *s*), and the independent samples t-test was used for the comparison between the two groups, and the paired samples t-test was used for the comparison before and after surgery in the same group. For outcomes measured at multiple postoperative time points (VAS scores, ODI, and inflammatory markers), repeated-measures analysis of variance (ANOVA) was employed to evaluate the overall time effect and group-by-time interaction. When a significant overall difference was detected, *post-hoc* pairwise comparisons were performed using the Bonferroni correction for multiple comparisons. The counting data were expressed as rate (%), and the *χ*^2^ test was used for the comparison between the two groups. The rank sum test was used for the non-parametric test of the ranked data such as MacNab efficacy evaluation. *P* < 0.05 was considered to have a statistically significant difference.

## 3 Results

### 3.1 Analysis of the number of participants

**Figure 1:**
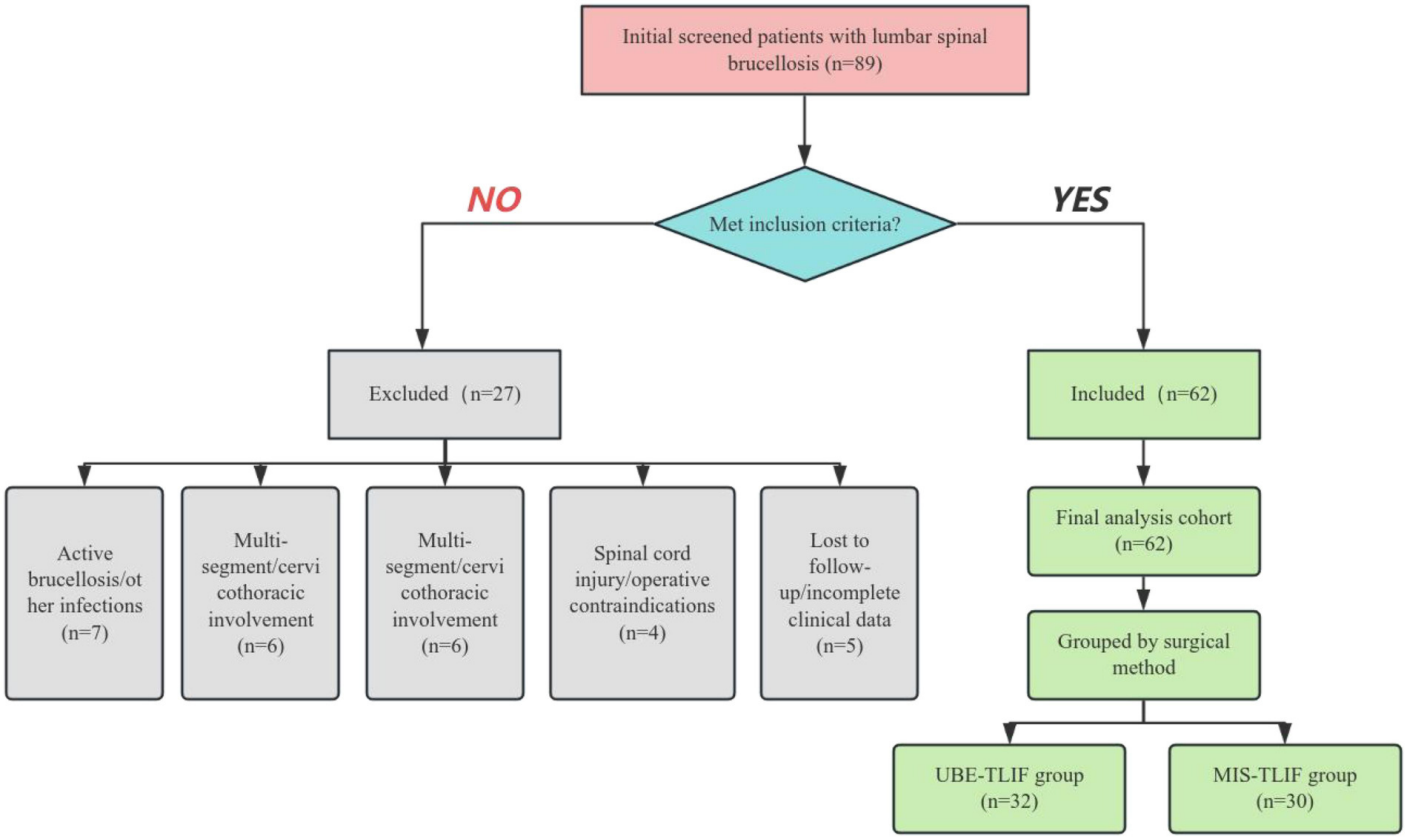
Flow chart of patient enrollment and grouping.

A total of 89 patients with lumbar spinal brucellosis were initially screened. Among them, 27 patients were excluded due to the following reasons: 7 with active brucellosis or other concurrent infections, 12 with multi-segment lumbar brucellosis or cervicothoracic involvement, 4 with spinal cord injury or surgical contraindications, and 5 with loss to follow-up or incomplete clinical data. Finally, 62 eligible patients were included in the analysis and divided into two groups according to surgical methods: 32 in the UBE-TLIF group and 30 in the MIS-TLIF group (Figure 1).

### 3.2 Comparison of baseline data

There were no statistically significant differences in gender, age, BMI, disease course, affected spinal segments, preoperative brucella agglutination titer, ESR, CRP, PCT, VAS scores of low back and leg pain and ODI between the UBE-TLIF group and the MIS-TLIF group (*P* > 0.05), and the baseline data were comparable (Table 1).

**Table 1:** Comparison of baseline data between the two groups.

Index	UBE-TLIF group (*n* = 32)	MIS-TLIF group (*n* = 30)	*t/χ^2^*	*P*
Gender (Male/Female, n)	20/12	18/12	0.105	0.746
Age (*x̅* *±* *s*, years)	48.56 ± 6.23	47.89 ± 5.98	0.428	0.670
BMI (*x̅* *±* *s*, kg/m^2^)	22.35 ± 2.11	22.68 ± 1.98	0.654	0.515
Disease course (*x̅* *±* *s*, months)	5.68 ± 2.15	5.92 ± 2.33	0.412	0.682
Affected segment (n, L1/L2/L3/L4/L5)	2/5/9/10/6	1/4/8/9/8	0.587	0.964
Brucella agglutination titer (*x̅* *±* *s*)	1:160.25 ± 35.68	1:158.67 ± 36.21	0.189	0.850
Preoperative ESR (*x̅* *±* *s*, mm/h)	78.56 ± 18.92	79.23 ± 19.56	0.135	0.893
Preoperative CRP (*x̅* *±* *s*, mg/L)	32.68 ± 8.56	33.15 ± 9.12	0.201	0.841
Preoperative PCT (*x̅* *±* *s*, ng/mL)	5.89 ± 1.23	5.98 ± 1.31	0.287	0.775
Preoperative low back pain VAS (*x̅* *±* *s*, points)	6.25 ± 0.89	6.33 ± 0.92	0.321	0.749
Preoperative leg pain VAS (*x̅* *±* *s*, points)	5.88 ± 0.78	5.95 ± 0.81	0.305	0.761
Preoperative ODI (*x̅* *±* *s*, %)	62.35 ± 5.68	63.12 ± 5.92	0.518	0.606

BMI, body mass index; ESR, erythrocyte sedimentation rate; CRP, c-reactive protein; PCT, procalcitonin; VAS, visual analog scale; ODI, Oswestry disability index. Data are presented as *n* or mean ± standard deviation.

### 3.3 Comparison of surgical indicators

The operative time, intraoperative blood loss, incision length and hospitalization time of the UBE-TLIF group were significantly shorter/lower than those of the MIS-TLIF group, and the differences between the two groups were statistically significant (*P* < 0.05) (Table 2).

**Table 2:** Comparison of surgical indicators between the two groups (x̅ ± s).

Surgical indicators	UBE-TLIF group (*n* = 32)	MIS-TLIF group (*n* = 30)	*t*	*P*
Operative time (min)	95.25 ± 15.68	138.67 ± 20.35	−9.025	<0.001
Intraoperative blood loss (mL)	65.38 ± 18.25	158.92 ± 30.56	−14.362	<0.001
Incision length (cm)	1.85 ± 0.32	3.68 ± 0.55	−16.895	<0.001
Hospitalization time (d)	7.25 ± 1.56	10.89 ± 2.13	−7.986	<0.001

UBE-TLIF, unilateral biportal endoscopy-transforaminal lumbar interbody fusion; MIS-TLIF, minimally invasive transforaminal lumbar interbody fusion.

### 3.4 Comparison of inflammatory indicators

The brucella agglutination titer, ESR, CRP and PCT of the two groups at 2 weeks, 1 month, 3 months and 6 months after surgery were significantly lower than those before surgery (*P* < 0.05); there were no statistically significant differences in the above inflammatory indicators between the two groups at the same postoperative time point (*P* > 0.05) (Table 3).

**Table 3:** Comparison of inflammatory indicators between the two groups (x̅ ± s).

Inflammatory indicators	Group	Preoperative	Postoperative 2 w	Postoperative 1 m	Postoperative 3 m	Postoperative 6 m
Brucella agglutination titer	UBE-TLIF (*n* = 32)	1:160.25 ± 35.68	1:85.38 ± 20.25	1:42.68 ± 10.35	1:25.89 ± 8.62	1:15.36 ± 5.25
	MIS-TLIF (*n* = 30)	1:158.67 ± 36.21	1:88.56 ± 22.36	1:45.25 ± 11.68	1:27.35 ± 9.18	1:16.89 ± 6.32
	*t/P*	0.189/0.850	0.605/0.547	0.892/0.376	0.586/0.560	0.925/0.359
ESR (mm/h)	UBE-TLIF (*n* = 32)	78.56 ± 18.92	45.38 ± 12.25	28.68 ± 8.35	18.92 ± 6.62	12.36 ± 4.25
	MIS-TLIF (*n* = 30)	79.23 ± 19.56	47.56 ± 13.36	30.25 ± 9.68	20.35 ± 7.18	13.89 ± 5.32
	*t/P*	0.135/0.893	0.685/0.495	0.652/0.517	0.725/0.471	0.986/0.328
CRP (mg/L)	UBE-TLIF (*n* = 32)	32.68 ± 8.56	18.38 ± 6.25	10.68 ± 3.35	6.92 ± 2.62	3.36 ± 1.25
	MIS-TLIF (*n* = 30)	33.15 ± 9.12	19.56 ± 7.36	11.25 ± 4.68	7.35 ± 3.18	3.89 ± 2.32
	*t/P*	0.201/0.841	0.658/0.512	0.528/0.600	0.568/0.572	0.956/0.343
PCT (ng/mL)	UBE-TLIF (*n* = 32)	5.89 ± 1.23	3.25 ± 0.89	1.86 ± 0.56	1.02 ± 0.32	0.56 ± 0.18
	MIS-TLIF (*n* = 30)	5.98 ± 1.31	3.48 ± 0.98	1.98 ± 0.62	1.15 ± 0.38	0.62 ± 0.21
	*t/P*	0.287/0.775	0.928/0.357	0.785/0.435	1.425/0.160	1.128/0.264

ESR, erythrocyte sedimentation rate; CRP, C-reactive protein; PCT, procalcitonin; 2w, 2 weeks; 1 m, 1 month; 3 m, 3 months; 6 m, 6 months.

### 3.5 Comparison of VAS scores and ODI

The VAS scores of low back and leg pain and ODI of the two groups at each postoperative time point were significantly lower than those before surgery (*P* < 0.05). The VAS scores of low back and leg pain in the UBE-TLIF group were significantly lower than those in the MIS-TLIF group at 1 week, 1 month and 3 months after surgery (*P* < 0.05), and there was no significant difference between the two groups at 6 months after surgery and the final follow-up (*P* > 0.05). There was no statistically significant difference in ODI between the two groups at each postoperative time point (*P* > 0.05) (Tables 4, 5).

**Table 4:** Comparison of VAS scores of low back and leg pain between the two groups (x̅ ± s, points).

VAS score	Group	Preoperative	Postoperative 1 w	Postoperative 1 m	Postoperative 3 m	Postoperative 6 m	Final follow-up
Low back pain	UBE-TLIF (*n* = 32)	6.25 ± 0.89	2.15 ± 0.68	1.58 ± 0.52	1.05 ± 0.36	0.82 ± 0.28	0.75 ± 0.25
	MIS-TLIF (*n* = 30)	6.33 ± 0.92	3.56 ± 0.89	2.68 ± 0.75	1.89 ± 0.58	0.88 ± 0.32	0.78 ± 0.28
	*t/P*	0.321/0.749	−6.895/<0.001	−6.025/<0.001	−6.586/<0.001	0.785/0.435	0.428/0.670
Leg pain	UBE-TLIF (*n* = 32)	5.88 ± 0.78	2.05 ± 0.62	1.48 ± 0.48	0.98 ± 0.32	0.75 ± 0.25	0.68 ± 0.22
	MIS-TLIF (*n* = 30)	5.95 ± 0.81	3.42 ± 0.85	2.55 ± 0.72	1.82 ± 0.55	0.82 ± 0.28	0.72 ± 0.25
	*t/P*	0.305/0.761	−7.025/<0.001	−6.258/<0.001	−6.892/<0.001	0.925/0.359	0.568/0.572

VAS, visual analog scale; 1w, 1 week; 1 m, 1 month; 3 m, 3 months; 6 m, 6 months.

**Table 5:** Comparison of ODI between the two groups (x̅ ± s, %).

Group	Preoperative	Postoperative 1 w	Postoperative 1 m	Postoperative 3 m	Postoperative 6 m	Final follow-up
UBE-TLIF (*n* = 32)	62.35 ± 5.68	38.56 ± 8.25	25.68 ± 6.35	18.92 ± 5.62	12.36 ± 4.25	10.89 ± 3.68
MIS-TLIF (*n* = 30)	63.12 ± 5.92	40.25 ± 9.36	27.25 ± 7.68	20.35 ± 6.18	13.89 ± 5.32	11.56 ± 4.12
*t/P*	0.518/0.606	0.785/0.435	0.892/0.376	0.725/0.471	0.986/0.328	0.658/0.512

ODI, Oswestry disability index; 1w, 1 week; 1 m, 1 month; 3 m, 3 months; 6 m, 6 months.

### 3.6 Comparison of intervertebral space height

The anterior and posterior intervertebral space height of the two groups at the final follow-up were significantly higher than those before surgery (*P* < 0.05); there was no statistically significant difference in the recovery rate of intervertebral space height between the two groups (*P* > 0.05) (Table 6).

**Table 6:** Comparison of intervertebral space height and recovery rate between the two groups (x̅ ± s, mm).

Index	Group	Preoperative	Final follow-up	Recovery rate (%)	*t/P* (recovery rate)
Anterior intervertebral space height	UBE-TLIF (*n* = 32)	4.25 ± 0.68	7.89 ± 0.92	85.68 ± 10.35	0.892/0.376
	MIS-TLIF (*n* = 30)	4.32 ± 0.72	7.68 ± 0.88	82.25 ± 11.68	
Posterior intervertebral space height	UBE-TLIF (*n* = 32)	3.85 ± 0.56	7.25 ± 0.85	88.36 ± 9.62	0.725/0.471
	MIS-TLIF (*n* = 30)	3.92 ± 0.62	7.02 ± 0.82	85.89 ± 10.18	

Recovery rate = (Final follow-up height - Preoperative height)/(Normal intervertebral space height - Preoperative height) × 100% (Normal lumbar intervertebral space height: anterior 8–10 mm, posterior 7–9 mm).

### 3.7 Comparison of postoperative complications

In the UBE-TLIF group, 3 cases of dural sac tear occurred. Among these, two cases were at the L4/5 segment and one at the L5/S1 segment. Intraoperatively, the dural tears were attributed to inadvertent mechanical injury during retraction of the dural sac under endoscopic visualization, given the narrow working corridor. All three cases were managed intraoperatively with placement of gelatin sponge and application of fibrin sealant; no conversion to open surgery was required. Postoperatively, patients were treated with bed rest for 5–7 days, with the drainage tube retained until drainage volume was <50 mL/day and no cerebrospinal fluid leakage was observed. All three patients recovered uneventfully without sequelae, and the mean time to symptom resolution was 6.3 ± 1.2 days.

In the MIS-TLIF group, 4 cases of nerve root traction injury occurred, involving the L4 nerve root in two cases, L5 nerve root in one case, and S1 nerve root in one case. Intraoperative findings suggested that the injuries were associated with excessive or prolonged retraction of the nerve root during focus debridement under the limited transforaminal visual field. Postoperatively, these patients received neurotrophic agents (methylcobalamin 0.5 mg intramuscularly once daily) and analgesic therapy (celecoxib 200 mg orally as needed). Symptoms of radicular pain and paresthesia gradually improved, with complete resolution achieved within 2 to 4 weeks (mean recovery time: 18.5 ± 5.3 days). No persistent neurological deficits were observed at final follow-up.

No complications such as cerebrospinal fluid leakage, incision infection, epidural hematoma, implant loosening, or brucellosis recurrence occurred in either group. The total complication rate was 9.38% (3/32) in the UBE-TLIF group and 13.33% (4/30) in the MIS-TLIF group, with no statistically significant difference (*χ*^2^ = 0.327, *P* = 0.568).

### 3.8 Comparison of long-term efficacy indicators

The mean follow-up time was 18.5 ± 3.6 months in the UBE-TLIF group and 18.1 ± 3.9 months in the MIS-TLIF group, with no significant difference between the two groups (*t* = 0.426, *P* = 0.671). At the final follow-up, all patients in the two groups achieved bone fusion, the bone fusion rate of the UBE-TLIF group was 96.88% (31/32) and that of the MIS-TLIF group was 93.33% (28/30), with no statistically significant difference (*P* > 0.05). The excellent and good rate of MacNab efficacy evaluation was 93.75% (30/32) in the UBE-TLIF group and 90.00% (27/30) in the MIS-TLIF group, and the difference between the two groups was not statistically significant (*P* > 0.05) (Table 7).

**Table 7:** Comparison of long-term efficacy indicators between the two groups [n, rate (%)].

Long-term efficacy indicators	UBE-TLIF group (*n* = 32)	MIS-TLIF group (*n* = 30)	*χ^2^*	*P*
Bone fusion grading (Grade 1/Grade 2)	31/1	28/2	0.385	0.535
Bone fusion rate(%)	96.88	93.33	-	-
MacNab efficacy evaluation (Excellent/Good/Fair/Poor)	20/10/2/0	18/9/3/0	0.287	0.866
Excellent and good rate (%)	93.75	90.00	-	-

Bone fusion was evaluated by lumbar spine CT at the final follow-up. Grade 1: solid fusion with trabecular bone crossing the graft area, stable intervertebral space, no cage subsidence or displacement, and no abnormal movement on dynamic radiography. Grade 2: uncertain or non-union. MacNab efficacy evaluation: excellent (no low back and leg pain, normal lumbar spine activity, no impact on daily life and work), good (occasional mild low back and leg pain, slight limitation of lumbar spine activity, little impact on daily life and work), fair (frequent low back and leg pain, obvious limitation of lumbar spine activity, great impact on daily life and work), poor (persistent severe low back and leg pain, loss of lumbar spine activity function, unable to take care of oneself).

### 3.9 Typical case

**Figure 2:**
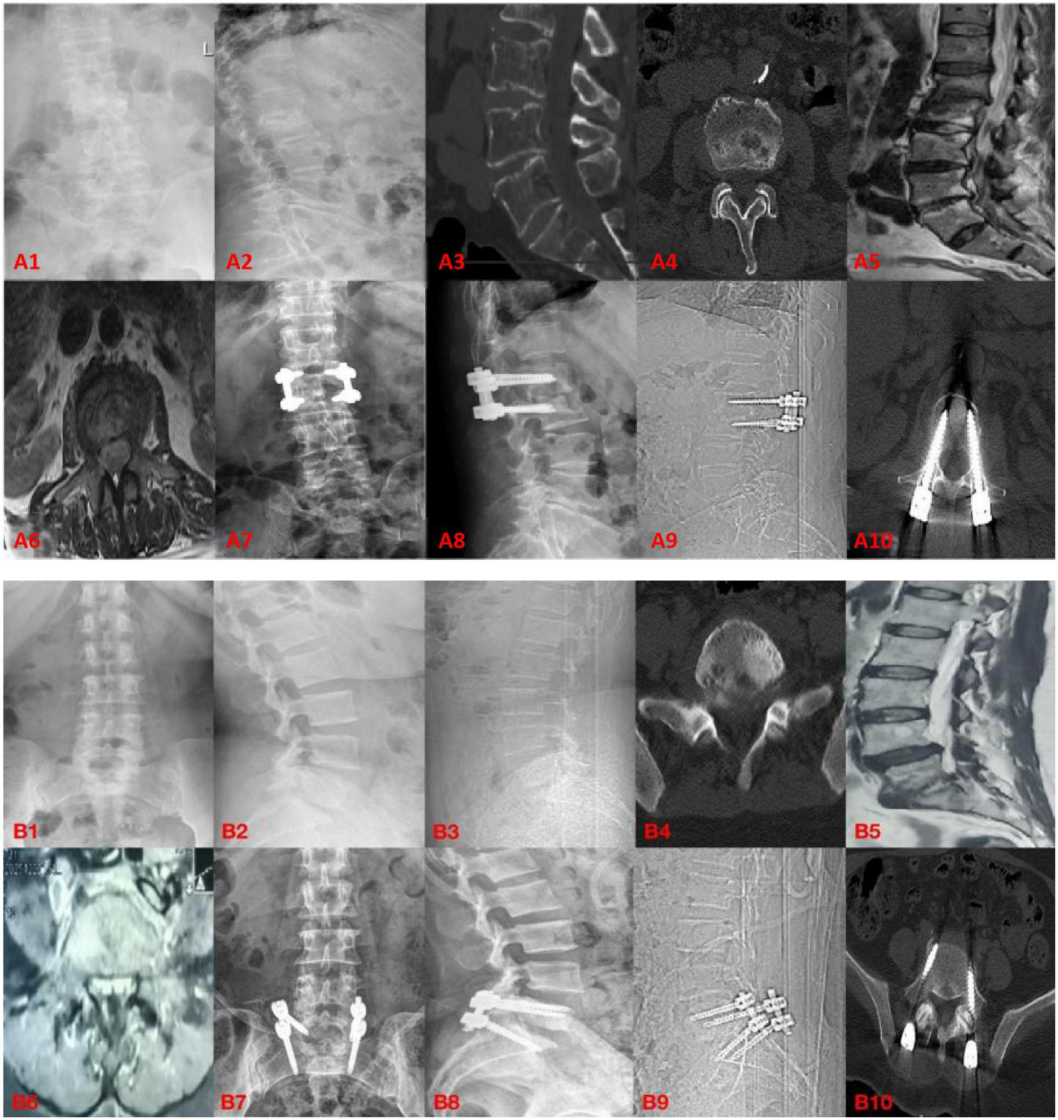
Imaging data of a typical case.

Patient 1 (MIS-TLIF group), male, 73 years old. Chief complaint: Low back pain associated with bilateral lower limb pain for 1 year. Upon admission, the patient presented with straightening of lumbar lordosis, tenderness over the spinous processes of T10–L3 and bilateral paravertebral muscles, severe limitation of lumbar motion, and hypesthesia in both lower limbs. Bilateral straight leg raise tests and crossed straight leg raise tests were positive. Preoperative lumbar x-ray, CT, and MRI showed bone destruction and intervertebral space narrowing at L2/3 (Figures 2A1–A6). The patient was diagnosed with lumbar tuberculosis (L2/3) and underwent MIS-TLIF under general anesthesia. Postoperatively, the patient's low back and bilateral lower limb pain were significantly relieved. At final follow-up, imaging (Figures 2A7–A10) showed stable internal fixation without loosening or breakage.

Patient 2 (UBE-TLIF group), female, 53 years old. Chief complaint: Low back pain associated with bilateral lower limb pain for 2 months, aggravated for 3 days. Upon admission, the patient presented with a limping gait, tenderness over the lumbosacral spinous processes, and mild hypesthesia in the posterolateral aspects of both lower limbs. Preoperative lumbar x-ray, CT, and MRI showed bone destruction and intervertebral space narrowing at L5 (Figures 2B1–B6). The patient was diagnosed with lumbar tuberculosis (L5) and underwent UBE-TLIF under general anesthesia. Postoperatively, the patient's low back and bilateral lower limb pain were significantly relieved. At final follow-up, imaging (Figures 2B7–B10) showed stable internal fixation without loosening or breakage.

## 4 Discussion

Lumbar spinal brucellosis is a common infectious spinal disease, which is the result of Brucella invading the lumbar vertebral body and intervertebral disc and causing persistent inflammatory reaction and bone destruction. The core of its treatment is to eliminate the infectious focus, control the inflammatory reaction, restore the spinal structure and stability, and relieve the nerve compression symptoms on the basis of standardized anti-brucellosis drug therapy (11–13). With the transformation of spinal surgery to minimally invasive, the application of MIS-TLIF and UBE-TLIF in lumbar spinal brucellosis has gradually become a research hotspot, but the comparative analysis of the two minimally invasive techniques is still lacking. This study expanded the sample size to 62 cases, and systematically compared the clinical efficacy of UBE-TLIF and MIS-TLIF in the treatment of lumbar spinal brucellosis, which confirmed the safety and effectiveness of the two methods, and further found the minimally invasive advantages of UBE-TLIF.

In this study, the baseline data of the two groups were comparable, and the results showed that the UBE-TLIF group had significant advantages in operative time, intraoperative blood loss, incision length and hospitalization time. The main reason is that UBE-TLIF constructs two small incisions of less than 1 cm to establish the operation channel, which avoids the large-scale stripping of paraspinal muscles in open surgery and even MIS-TLIF, and reduces the surgical trauma and intraoperative blood loss. At the same time, UBE-TLIF realizes 360° non-dead angle observation under endoscopic vision, which makes the focus debridement and pedicle screw implantation more precise, shortens the operative time, and thus accelerates the postoperative recovery of patients and shortens the hospitalization time (14). MIS-TLIF also has the characteristics of minimally invasive, but its incision length is longer than that of UBE-TLIF, and the paraspinal muscle separation range is larger, so the surgical trauma and intraoperative blood loss are relatively increased, and the postoperative recovery time is slightly longer (15–17).

In terms of inflammatory control, the brucella agglutination titer, ESR, CRP and PCT of the two groups decreased significantly after surgery, and there was no significant difference between the two groups. This indicates that both UBE-TLIF and MIS-TLIF can achieve thorough debridement of lumbar spinal brucellosis focus under the premise of standardized anti-brucellosis drug treatment, eliminate the source of infection, and effectively control the systemic and local inflammatory reaction. The key to effective inflammatory control in the two surgical methods is the thorough debridement of the focus: MIS-TLIF debrides the focus through the transforaminal approach under direct vision, while UBE-TLIF can more clearly observe the scope of the focus under endoscopic magnification, and thoroughly remove the necrotic bone tissue and inflammatory granulation tissue in the spinal canal and intervertebral space through the flexible operation of instruments in the working channel, and the repeated flushing of the surgical field with antibiotic-containing normal saline further eliminates the residual Brucella and inflammatory factors, so the two methods have the same effect on inflammatory control (18–21).

The comparable outcomes between the two techniques in inflammatory control, long-term functional recovery, and bone fusion rate can be attributed to several common factors. First, both UBE-TLIF and MIS-TLIF enable thorough debridement of the infectious focus—the former through a magnified endoscopic view and the latter via direct transforaminal visualization—supplemented by intraoperative irrigation with rifampicin-containing saline to eliminate residual pathogens. Second, both procedures achieve reliable spinal stability reconstruction using interbody bone grafting combined with bilateral pedicle screw fixation, which provides a favorable mechanical environment for bone fusion. Third, all patients received standardized, full-course anti-brucellosis pharmacotherapy, which serves as the cornerstone of infection control. Thus, when thorough debridement, stable fixation, and appropriate antibiotic therapy are uniformly achieved, both minimally invasive techniques yield satisfactory and comparable core efficacy outcomes.

Pain relief and functional recovery are important indicators to evaluate the clinical efficacy of lumbar spinal brucellosis surgery. The results of this study showed that the VAS scores of low back and leg pain in the UBE-TLIF group were significantly lower than those in the MIS-TLIF group at the early postoperative stage (1 week, 1 month, 3 months), and the differences disappeared at the late postoperative stage. This is closely related to the different degrees of surgical trauma of the two methods: UBE-TLIF causes minimal damage to paraspinal muscles, ligaments and other soft tissues, and the inflammatory reaction and pain caused by surgical trauma are mild in the early postoperative stage; while MIS-TLIF has a relatively large range of paraspinal muscle separation, and the postoperative muscle edema and inflammatory reaction are more obvious, resulting in relatively severe early pain (18, 22). With the gradual recovery of soft tissue injury, the pain difference between the two groups gradually disappears at 6 months after surgery. In terms of lumbar spine function recovery, the ODI of both groups was significantly improved after surgery, and there was no significant difference between the two groups, indicating that both surgical methods can effectively decompress the nerve root, restore the spinal structure and stability, and achieve good functional recovery effects.

The restoration of intervertebral space height and bone fusion are the key to maintaining long-term spinal stability and avoiding disease recurrence. The results showed that the intervertebral space height of the two groups was significantly restored after surgery, and the bone fusion rate was high at the final follow-up, with no significant difference between the two groups. This is because both UBE-TLIF and MIS-TLIF adopt the method of intervertebral bone graft fusion combined with pedicle screw internal fixation, which can effectively restore the intervertebral space height and the physiological curvature of the lumbar spine, and provide a stable mechanical environment for bone fusion. UBE-TLIF prepares the bone graft bed under endoscopic magnification, which can ensure the full exposure of the fresh bone surface and improve the bone fusion rate; MIS-TLIF has a wider surgical operation space, which is conducive to the implantation of bone graft and cage, and also can achieve good bone fusion (2, 23). At the final follow-up, there was no recurrence of brucellosis or implant-related complications in either group, which indicated that the two surgical methods have good long-term safety and effectiveness.

In terms of complications, the main complications of the UBE-TLIF group were dural sac tear, and the main complications of the MIS-TLIF group were nerve root traction injury, all of which recovered after conservative treatment, and the total complication rate of the two groups was not significantly different. The dural sac tear in the UBE-TLIF group is mainly related to the narrow surgical channel and the need for careful retraction of the dural sac during endoscopic operation, which requires the surgeon to have proficient endoscopic operation skills (24); the nerve root traction injury in the MIS-TLIF group is mainly due to the limited surgical field of view, which is easy to cause accidental traction of the nerve root during focus debridement (25). This suggests that regardless of which surgical method is adopted, the surgeon must master the corresponding surgical skills, standardize the operation steps, and reduce the occurrence of complications. In addition, no incision infection or epidural hematoma occurred in this study, which is related to the minimally invasive trauma of the two methods, thorough intraoperative hemostasis and postoperative standardized anti-infection treatment.

Impact of the learning curve on study results It is important to acknowledge that the learning curve for UBE-TLIF is relatively steep compared with MIS-TLIF. Previous studies have reported that the learning curve for UBE-TLIF typically requires approximately 30–40 cases to achieve stabilization of operative time and reduction of complications. In the present study, all UBE-TLIF procedures were performed by two surgeons who had each completed over 50 independent cases prior to the study period, thereby placing them well beyond the learning curve plateau. Therefore, the favorable outcomes observed in the UBE-TLIF group—including shorter operative time, reduced blood loss, and acceptable complication rates—reflect the performance of experienced surgeons rather than the early learning phase. However, these findings may not be directly generalizable to surgeons in the early stages of adopting UBE-TLIF. For MIS-TLIF, the three participating surgeons had each performed over 80 cases prior to the study, placing them well beyond the learning curve for this technique as well. Thus, the comparative results reflect the performance of experienced surgeons for both techniques, minimizing learning curve-related bias.

It should be noted that this study has several limitations. First, this study is a single-center retrospective study with inherent selection bias in patient enrollment, surgical technique allocation, and outcome assessment. The lack of randomization is a major limitation that precludes causal inference regarding the comparative efficacy of the two surgical techniques. Although we have provided detailed information on the decision-making process for technique selection, unmeasured confounders may still exist.Second, the sample size, while sufficient to detect differences in the primary outcome (operative time), may be underpowered for secondary outcomes such as complication rates, which have lower expected incidences. Therefore, the absence of statistically significant differences in complication rates should be interpreted with caution. Third, the follow-up duration is limited, and longer-term outcomes and late complications require further observation. Fourth, the inclusion criteria were restricted to patients with single-segment lumbar spinal brucellosis (L1–L5), and patients with multi-segment involvement or combined thoracic/cervical lesions were explicitly excluded. This substantially limits the generalizability of our findings to more complex cases. In clinical practice, a considerable proportion of patients with spinal brucellosis present with multi-level involvement, which often requires more extensive debridement, longer segment fixation, and potentially different surgical strategies. Therefore, the favorable outcomes observed in this study may not be directly applicable to patients with multi-segment disease, and caution should be exercised when extrapolating these results to such cases. Future studies specifically addressing multi-level lumbar spinal brucellosis are needed to determine whether the advantages of UBE-TLIF observed in single-segment cases can be similarly achieved in more complex scenarios.Fifth, this study did not analyze the impact of patients’ nutritional status or underlying comorbidities (such as diabetes mellitus or chronic kidney disease) on inflammatory control and bone fusion, factors that may influence postoperative recovery. Sixth, multivariate regression analysis was not performed to identify independent risk factors affecting therapeutic efficacy, which limits the ability to control for potential confounders. Seventh, although all participating surgeons exceeded the defined proficiency thresholds for both techniques, the results may not be generalizable to surgeons in the early learning phase of UBE-TLIF, and the learning curve remains a potential confounding variable that could influence outcomes in broader clinical practice.Future multi-center, prospective, large-sample randomized controlled trials are needed to further validate the clinical efficacy and safety of UBE-TLIF in lumbar spinal brucellosis and to establish individualized surgical strategies for patients with different disease characteristics.

In conclusion, both UBE-TLIF and MIS-TLIF are safe and effective minimally invasive surgical methods for the treatment of lumbar spinal brucellosis, which can effectively control inflammatory reaction, relieve pain, restore spinal function and achieve good long-term bone fusion. Compared with MIS-TLIF, UBE-TLIF has more obvious minimally invasive advantages in shortening operative time, reducing intraoperative blood loss, minimizing surgical trauma and relieving early postoperative pain, and is worthy of clinical priority promotion and application on the premise that the surgical team masters proficient endoscopic operation skills. For patients with extensive lumbar spinal brucellosis lesions and insufficient endoscopic operation skills of the surgeon, MIS-TLIF is still a reliable surgical option.

## Data Availability

The raw data supporting the conclusions of this article will be made available by the authors, without undue reservation.
